# Annual Commencement of the Baltimore College of Dental Surgery

**Published:** 1853-04

**Authors:** 


### EDITORIAL DEPARTMENT.

*Annual Commencement of the Baltimore College of Dental Surgery.*—The thirteenth commencement of the above named institution was held on the evening of the 25th of March, 1853. At an early hour the saloon of the new college building was crowded with ladies and gentlemen, to witness the ceremony of conferring the degree of Doctor of Dental Surgery upon the graduating class.

The exercises were opened with prayer by the Rev. Mr. Smith, of the Presbyterian church. The names of the graduates, their residences and subjects of theses, were then announced by the Dean, Professor W. R. Handy. They are as follows:

1. B. F, Arrington, M. D., N. C. Constitutional treatment of odontalgia.
2. A. B. Arthur, N. Y., Diseases of the inferior maxilla from diseased teeth.
3. A. J. Brown, Md., Physical marks of the teeth.
4. S. T. Church, M. D., Md., Formation of the teeth.
5. S. J. Cockerille, Va., Anatomy—its progress.
6. C. R. Coffin, Maine, Artificial teeth.
7. G. L. Cook, Massachusetts, Neuralgia of the face.
8. M. D. French, " Necrosis of the superior maxilla.
9. F. M. Green, M. D., Mississippi, Caries of the teeth.
10. D. P. Gregg, Virginia, Structure of the salivary glands.
11. G. P. Kingsley, Massachusetts, Artificial teeth.
12. F. F. E. Kirchner, Maryland, Mechanical dentistry.
13. S. A. McDowell, Pennsylvania, First dentition—morbid effects of.
14. T. C. Royce, N. York, Pure tin as a base for artificial teeth.
15. B. S. Reilly, M. D., District of Columbia, First dentition.
16. A. J. Sedwick, Virginia, Indications for filling teeth.
17. D. J. Shelton, " Filling teeth.
18. W. C. Stewart, Maryland, Caries of the teeth.
19. M. S. Taylor, Virginia, Progress of dental surgery.
20. R. H. Tucker, Bermuda, Filling teeth.
21. D. Tracy, Massachusetts, Diseases of the dental pulp.
22. H. N. Wodsworth, District of Columbia, Absorption of saliva from the cavity of a tooth preparatory to filling.

The authority for conferring the degree came next in order, and was read by the Dean. The graduating class were then requested to rise, when they were asked by Professor Harris, if they were willing to promise to hold no secrets, to discountenance quackery of every kind pertaining to the profession into which they were about to be inducted, to do all in their power to advance the science and art of dental surgery, and to elevate the dignity of their calling? Having signified their assent to these requirements, the class resumed their seats; and were called up by threes in succession, and the degree conferred upon them by Professor Harris; the intervals were enlivened by appropriate music. As the students received their diplomas they were greeted with showers of boquets.

The ceremony of conferring the degree being over, Professor Austen pronounced a very able Valedictory, which will be found in another part of the Journal. Dr. Gregg, on behalf of the class, responded, returning thanks for the instruction and kindness which they had received at the hands of the faculty during the past session.

Dr. W. H. Dwinelle, of Cazenovia, N. Y., read a short but beautiful poetical address, containing much wholesome advice, and in which he gave a graphic description of the bungling dentist, as well as a very touching episode to the memory of the late Dr. Cleaveland, of Charleston, S. C.

Professor Bond then announced that an eminent dentist of the state of New York, whose name he was not permitted to mention, had presented to the faculty fifty dollars, to be awarded at the close of the next session in the form of a medal or otherwise, to the student who should present the best thesis. This act of liberality, by a gentleman who is only known to most of the faculty by reputation, for the promotion of dental education, bespeaks, we think, the dawn of a brighter day and a more brilliant future to the science and art of dental surgery.

The operations performed in the infirmary of the college during the last session are as follows:

| | |
|---|---|
| Teeth filled, in which caries had not penetrated to the pulp cavity, | 481 |
| Separations made between teeth, . . . . . . . | 138 |
| Teeth extracted, . . . . . . . . . . | 400 |
| Cases from which salivary calculus was removed, . . . | 64 |
| " of diseased gums successfully treated, . . . . . | 60 |
| Teeth successfully filled over exposed nerve, . . . . . | 19 |
| " " " into pulp cavity and roots, . . | 9 |
| Pivot teeth inserted, . . . . . . . . . . . | 7 |
| Artificial teeth mounted on plate, . . . . . . . | 880 |
| Operations on diseased maxillæ, . . . . . . . | 1 |
| | 2,059 |

Among the professional gentlemen from abroad who were present, at the commencement, were the professors of the Philadelphia College of Dental Surgery.

The class of the session just closed, numbered forty-six, fifteen more than ever matriculated at any preceding session.

